# SNHG6 203 RNA May be Involved in the Cell Cycle Progression in HER2-Negative Breast Cancer Cells

**DOI:** 10.30699/IJP.2022.525346.2607

**Published:** 2022-08-11

**Authors:** Amin Jafari-Oliayi, Shahriar Dabiri

**Affiliations:** *Pathology and Stem Cell Research Center, Department of Pathology, Afzalipour Medical School, Kerman University of Medical Sciences, Kerman, Iran*

**Keywords:** Breast neoplasm, Cell cycle, lncRNA

## Abstract

**Background & Objective:**

Long noncoding RNAs (lncRNAs) as challenging molecules are more known than those in the last decade. These transcripts have been validated for carcinogenesis in many types of tissue. Functions of lncRNAs in cancer induction include cell cycle, epithelial to mesenchymal transition progression, apoptosis inhibition, cell migration, and invasion stimulation. LncRNA small nucleolar host gene 6 (SNHG6) have been proven as an oncogenic transcript in many types of cancer.

**Methods:**

RNA extraction was performed for 47 breast specimens in patients with cancer and cDNAs were synthesized. Relative expression of target variants was determined by qPCR and calculated based on the ΔΔCt method. SNHG6 203 was cloned into pcDNA 3.1+ vector for overexpression in MCF7 (HER2-) and SK-BR3 (HER2+) cells. The cell cycle progression of transfected cells was assessed by flow cytometry. Cell migration ability of transfected cells was evaluated by the scratch method and Image J software. Finally, cell viability was assessed by the MTT method.

**Results:**

Among four splice variants of SNHG6 (202, 203, 204, and 207), SNHG6 203 was proved as an overexpressed splice variant in breast tumors. This transcript was expressed in HER2-negative breast tumors more frequently than in the positive ones. Overexpression of this variant in target cells resulted in cell cycle progression of MCF7 as HER2-negative cells. Moreover, the overexpression of SNHG6 203 led to lower migration ability of MCF7 cells and a non-significant reduction of their viability as HER2-negative breast cancer cells.

**Conclusion:**

Our results revealed that SNHG6 203 may be involved in the carcinogenesis of HER2-negative breast cancers via cell cycle progression.

## Introduction

Long noncoding RNAs (lncRNAs) were known as key transcripts in cellular mechanisms (1). Noncoding transcripts, such as lncRNAs, were considered the products of junk DNA, but nowadays, they are known as important members of the molecular system of the cells (2). LncRNAs, known as transcripts, have more than 200 nucleotides in length (3). They form secondary and tertiary structures in the aqueous environment of the cell (4) and may be involved in the carcinogenesis process (5). For example, HOTAIR lncRNA was proven in breast tissue carcinogenesis (6) and MALAT1 can induce cancer in the bladder and colorectal tissues (7).

Small nucleolar host genes (SNHGs) are the genes that contain small nucleolar RNA sequences. SNHG6, as an important member of this family, has been focused in cancer research in the last 5 years (8-10). SNHG6 has a short sequence in its gene called U87; therefore, another name for SNHG6 is the U87 host gene (11). U87 is a small nucleolar RNA (snoRNA) that guides the chemical modifications of some RNAs, such as snRNAs. This snoRNA guides the methylation complex to the target nucleotides that should be methylated. Nucleotide methylation is a common modification for ribosomal RNA (rRNAs). The rRNA modification is a critical step of ribosome biogenesis (12, 13). Every factor that affects this step may influence the translational fidelity. In addition, rRNA modification may affect the tendency of the ribosome to some messenger RNA (mRNA) for translation (14). 

SNHG6 was proved as an oncogenic RNA and is involved in cancer progression, such as breast (15), brain (16), prostate (17), or lung cancers (18). This transcript is located in both cytoplasm and nucleus, but mainly in the cytoplasm. Oncogenic characteristics of SNHG6 could originate from miRNA sponging ability (19). Moreover, the methylation complex may be recruited by this noncoding RNA (10). SNHG6 and many miRNAs are interconnected and this phenomenon affects many molecular mechanisms of the cells (8, 20-22). As a splice variant of the SNHG6 gene, SNHG6 203 has been proven as an oncogenic transcript in hepatocellular carcinoma (23). SNHG6 expression occurs in many cell lines and tissues, but the expression pattern of its variants has not been determined. Splice variants expression pattern of lncRNAs may be important for better prognosis and diagnosis of cancer (24). The prognosis of cancer is a vital step in cancer treatment. Therefore, every factor that enhances and assures cancer prognosis is crucial. In this study, the possible role(s) of SNHG6 203, a splice variant of the SNHG6 gene, in HER2-negative breast cancer carcinogenesis was investigated.

## Material and Methods

**Sample Collection**

A total of 94 breast cancer tumor and non-tumor tissues were collected from 47 patients with different variants of breast cancer. The samples and their data sheets were received from the Iran National Tumor Bank founded by the Cancer Institute of Tehran University of Medical Sciences for cancer research. Table 1 demonstrates some clinicopathological characteristics of the studied patients. 

**RNA Extraction**

RNA extraction was performed by RNX plus solution (Cinnagen, Iran). First, the sample was frozen using liquid nitrogen and the frozen tissue was ground for several minutes with 1 mL of RNX solution. The ground tissue was transferred to a microtube. Next, 200 µL of chloroform (Merck, Germany) was added to each microtube, and the microtubes were shaken vigorously and placed on ice for 15 min. Afterward, microtubes were centrifuged at 12000 RPM and 4ºC for 15 min. The upper transparent solution was transferred to a new microtube and was mixed gently with 500 µL of isopropyl alcohol (Merck, Germany) for RNA precipitation. After 40 min, microtubes were centrifuged at 12000 RPM and 4ºC for 15 min. The RNA pellets were observed in the bottom of microtubes. The RNA pellets were dissolved in 30 µL of nuclease-free water.

**Synthesis of cDNA**

Of the extracted RNA, 1 µg was used for the synthesis of cDNA. Extracted RNA, 200U DNase enzyme (Thermo Fisher Scientific, USA), and 1 µL of DNase buffer were mixed in a 10 µL volume reaction. After 30 min at 37ºC, co-purified DNA was degraded completely. The other steps were completed as performed before (24).

**RT-qPCR**

Relative gene expression was assessed by quantitative real-time PCR and calculated by the delta-delta CT method. We measured the expression level of target splice variants in all tumor samples and the expression level was compared with the β-Actin expression level of the same sample. The relative expression level of each sample was calculated based on the difference between the cts of the target variant and β-Actin. β-Actin is a housekeeping gene used as a reference gene for assessing gene expression levels in biological studies. The relative expression level of genes may be measured and compared by this method, known as the ΔΔct method (25). We designed primers based on primer design rules utilizing the GeneRunner software. Moreover, we used ABI real-time instrument (Life technology, USA) and its software for the measurement of relative gene expression. We conducted each assay in duplicate. The sequence of designed primers is mentioned in Table 2. In order to assess the relative expression of 4 target variants of SNHG6 1 µL cDNA, 5 µL of real-time master mix (Ex taq2 Takara, Japan), and 1 µL of reverse and forward primers in a 10 µL reaction were applied. The expression of targets was normalized by β-Actin as the reference gene. The cycling programs of primers are noted in Table 3. 

**Plasmid Construction and Cell Transfection**

The SNHG6 203 was cloned in pcDNA 3.1+ as a shuttling vector. The target sequence with cut sites for the digestion of Zho1 and BamH1 restriction enzymes (Takara, Japan) was amplified by PCR. The target sequence was visualized on agarose gel and then was purified by a DNA gel extraction kit (Sinaclon, Iran). The vector and insert were digested with restriction enzymes separately and were ligated together overnight at 14ºC. The construct was transformed into a proper bacterial host (DH5α) by the heat shock transformation method. Briefly, after a short incubation on ice, a mixture of chemically competent bacteria and DNA was placed at 42°C for 45 sec (heat shock) and was then placed back on ice. The SOC medium was added and the transformed cells were incubated at 37°C for 30 min with agitation. This traditional protocol can be used successfully to transform the most available competent bacteria. Finally, the construct was extracted through a mini-prep plasmid extraction kit (Bioneer, South Korea). For transfecting target breast cancer cells (MCF7 & SK-BR3), 2.5 µg of construct and 5 µL of lipofectamine 2000 (Invitrogen Company, USA) were mixed and the cells were exposed to transfection complex for 5 h. Next, the mixture of medium and transfection complex was removed and a fresh medium containing 10% FBS (Gibco, New Zealand) was added. 

**Cell Culture**

This research studied SNHG6 203 expression in breast cancer patients. In addition, the influence of SNHG6 203 on some behaviors of HER2-negative breast cancer cells (MCF7 ER+, PR+/–, and HER2–) and HER2-positive breast cancer cells (SK-BR3 ER-, PR–, and HER2+) was investigated. These cell lines were different in terms of HER2 status. Moreover, many HER2-negative patients in this study and MCF7 cells had the same hormonal status (ER & PR positive). The MCF7 and SK-BR-3 cells were cultured at 37ºC and 5% CO_2_ condition in a humid atmosphere of a Memmert incubator (INC246MED115V, CO_2_ Incubators, INCOmed, Memmert). Also, 10% of fetal bovine serum and RPMI 1640 and DMEM media were used (Gibco) for MCF7 and SK-BR-3 cells, respectively.

**Cell Cycle Analysis**

The cell cycle was analyzed to determine cell fractions in different phases of the cell cycle. The cells were prepared and analyzed by flow cytometry (Partec, Germany) as conducted before (26). Cell cycles of the cells that hold SNHG6 203 construct were compared with the cycle of the cells containing intact pcDNA3.1+ plasmid (as control cells). 

**Scratch Wound Healing Assay**

To assess the migration ability of target cells, a wound-healing assay was performed. First, control and SNHG6 203 overexpressing cells were permitted to grow and cover the surface of the plate completely. Next, a scratch with a yellow tip was generated. Afterwards, the cells were allowed to migrate across the scratch, wound closure was photographed, and the images were analyzed by Image J software (Rasband, W.S., ImageJ, U.S. National Institute of Health, Bethesda, Maryland, USA, https://imagej.nih.gov/ij/, 1997-2018.). The migration of control cells was considered as 100 and the migration of SNHG6 203 overexpressing cells was calculated relative to that. The data were statistically analyzed using the unpaired t-test. 

**Cell Viability Assay (MTT)**

Cell viability was evaluated utilizing the MTT assay. The control and SNHG6 203 overexpressing cells were cultured in a 96-well plate and the viability of the cells was determined 24, 48, and 72 h after transfection. The protocol was completed according to Keshavarz and Asadi (27).

**Statistical Analysis**

Statistical analyses were performed by the unpaired t-test and two-way analysis of variance (ANOVA) using the GraphPad Prism software version 6. P<0.05 was considered significant**.**

## Results

**SNHG6 203 was Frequently Expressed in HER2-negative Breast Cancers.**

**Fig. 1:**
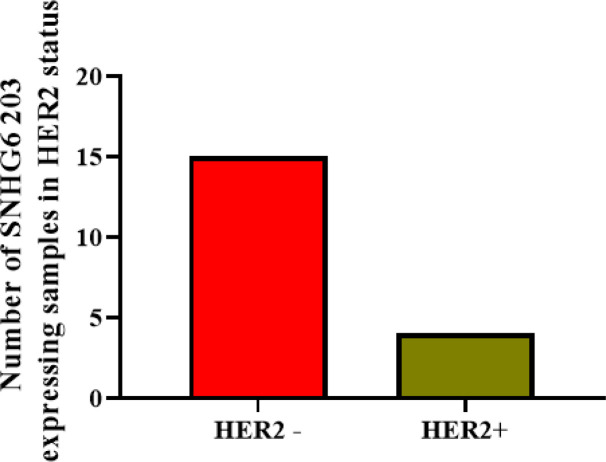
The number of HER2 negative and HER2 positives patients who presented with detectable expression of SNHG6 203 in their tumors

The expression data of breast cancer patients revealed that SNHG6 203 was expressed more frequently in HER2-negative breast tumors than in HER2-positive cases (Figure 1 and Table 4).

**SNHG6 203 Was More Expressed in Low-Grade Breast Tumors than in High-grade Tumors.**

**Fig. 2:**
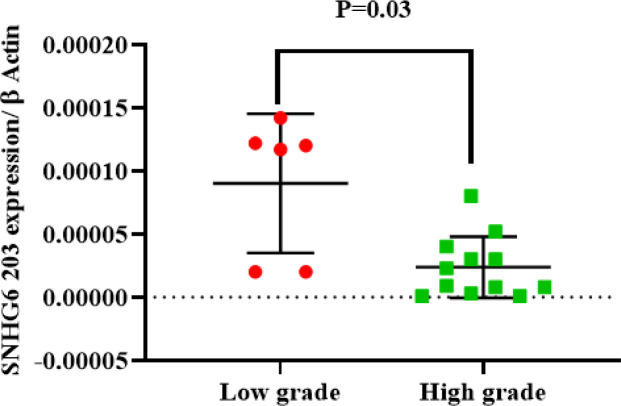
Significant higher SNHG6 203 expression in low grade breast tumors compared to high grade tumors

Statistical analysis (unpaired t-test) of q-PCR results demonstrated that SNHG6 203 was more expressed in the low-grade breast tumor tissues compared to in high-grade ones (Figure 2). 

**Fig. 3:**
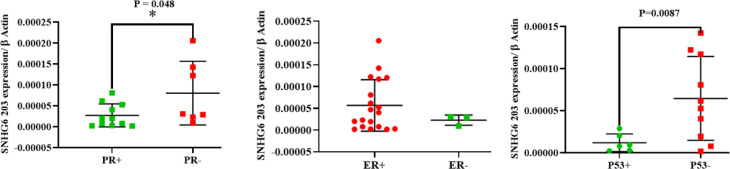
SNHG6 203 was expressed significantly in P53 and progesterone negative breast tumors more than the more positives

**SNHG6 203 was Upregulated in P53- and Progesterone-negative Breast Tumor Tissues compared to the Positive cases**

The SNHG6 203 expression level measurement in patients with different P53, progesterone, and estrogen statuses revealed that SNHG6 203 was expressed in P53- and progesterone-negative breast tumors more than the positive ones (Figure 3). The unpaired t-test was used for statistical analysis. 

**SNHG6 203 Overexpression was More Effective on the Cycle Progression of the MCF7 Cells Compared to the SK-BR3 Cells.**

**Fig. 4:**
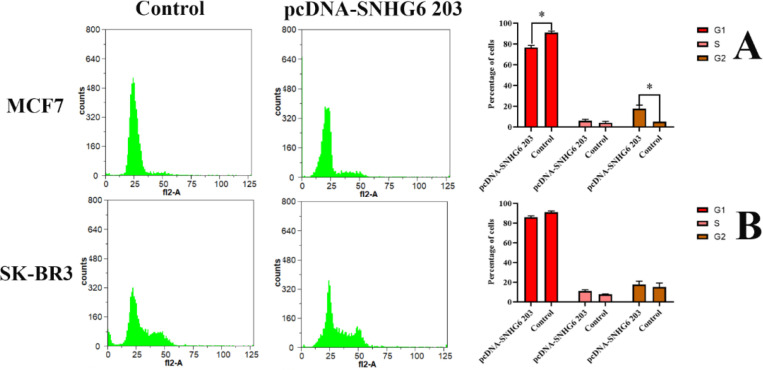
SNHG6 203 over expression in target cell lines led to G1 progression in MCF7 cells as HER2 negative breast cancer cells

Transfection of MCF7 and SK-BR3 cells with SNHG6 203 construct and statistical analysis by the unpaired t-test showed that the G1 progression of MCF7 cells occurred more noticeably than SK-BR3 cells following the SNHG6 203 overexpression. The difference was significant (Figure 4).

**Migration Ability of MCF7 Cells Reduced More than SK-BR3 Cells Following SNHG6 203 Overexpression.**

**Fig. 5:**
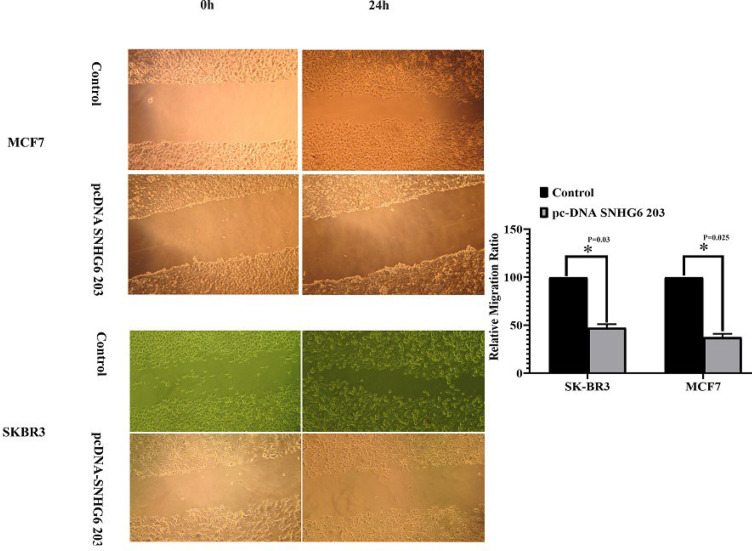
SNHG6 203 overexpression led to decreased migration ability of both cell lines compared to the controls

The SNHG6 203 overexpression in the target breast cancer cell lines led to a lower migration ability in overexpressing cells than their counterpart control cells. The SK-BR3 cells showed a higher migration ability compared to MCF7 cells when SNHG6 203 was overexpressed (Figure 5). The unpaired t-test was used for statistical analysis. 

**SNHG6 203 Overexpressing Cells Showed Different Cell Viability.**

**Fig. 6:**
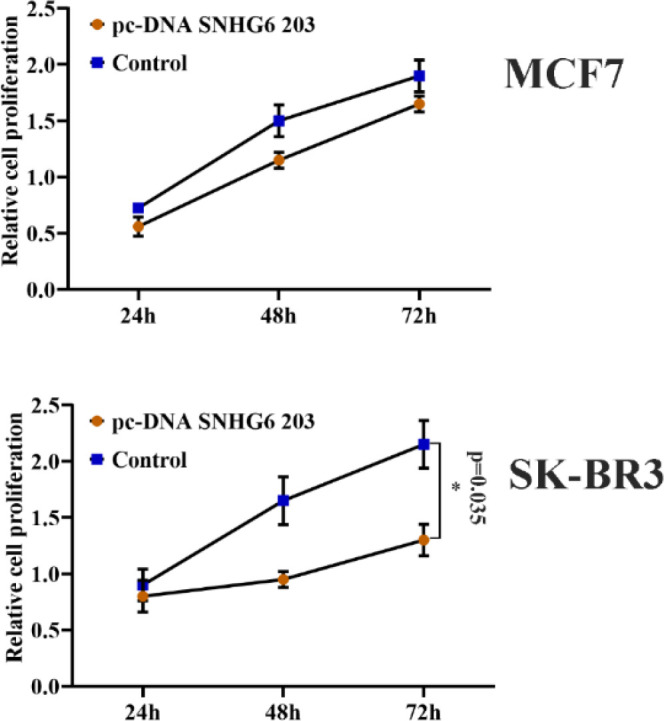
SNHG6 203 overexpression led to less viability of SKBR3 cells compared to the MCF7 cells

Overexpressing MCF7 cells indicated an insignificant difference in cell viability compared to their relative control. Following SNHG6 203 overexpression, SK-BR3 cells demonstrated a significant reduction in viability compared to their counterpart controls (Figure 6). Two-way ANOVA test was used.

**Table 1:** Status of hormone receptors (ER and PR), HER2, P53, and different grades

Characteristics	Number
ER status ER – ER +	**13** **28**
PR status PR – PR +	**18** **23**
HER2 status HER2 – HER2 +	**31** **8**
P53status P53 – P53 +	**22** **14**
Grade High Grade Low Grade	**13** **27**

**Table 2:** Sequence of the used primers

Gene	Primer sequence 5’>3’
SNHG6 203 F	**GAGTGCCTAAGAGCTGTCTTCC**
SNHG6 203 R	**GCCGCGTGATCCTAGTAGTT**
SNHG6 203 cloning F	**atgatGGATCCGAGTGCCTAAGAGCTGTCTTCC**
SNHG6 203 cloning R	**atgtaCTCGAGCAGCCTCAATGAGAATCACCACC**
SNHG6 202 F	**CCAGTGCTTTGCAGTCAGGATTC**
SNHG6 202 R	**GCCGCGTGATCCTAGTAGTT**
SNHG6 207 F	**AAAACTACTAGGATCACGC**
SNHG6 207 R	**CTAGTGACTATGAGAATGGAG**
SNHG6 204 F	**CGCGAAGAGCCGTTAGTCAT**
SNHG6 204 R	**AAGTCATCATTGTGCCAGC**
β Actin F	**ACTCTCTTCCAGCCTTCCTTCCT**
β Actin R	**ACTGACAGCACTGTGTTGGCGTA**

**Table 3:** Conditions of the performed polymerase chain reactions

	Initial denaturation	Cycle denaturation	Annealing and extension	Number of Cycle	Annealing temperature
Β-Actin	40 s	5 s	30 s	40	**60ºC**
SNHG6 207	40 s	5 s	30 s	40	**65ºC**
SNHG6 202	40 s	5 s	40 s	40	**62ºC**
SNHG6 203	40 s	5 s	40 s	40	**62ºC**
SNHG6 204	40 s	5 s	42 s	40	**60ºC**
SNHG6 203 cloning primers	4 min	40 s	Annealing 35 s Extension 40 s	35	**60ºC**

**Table 4:** Significant expression data of some clinicopathological characteristics of the patients

Characteristics	Number (%)	Expression mean±SD	P-value
Her2 status Positive Negative	5 (10.6%) 15 (31.8%)	0.0000215±0.0000014 0.00005625±0.00000436	**0.03**
Grade High Grade Low Grade	12 (25.4%) 6 (12.7%)	0.000023±0.0000024 0.000009±0.000005	**0.03**
P53 status P53 positive P53 negative	6 (12.7%) 10 (21.3%)	0.000018±0.000010 0.000064±0.000049	**0.008**

## Discussion

The lncRNAs, as regulating molecular factors, appeared more interesting than ever before. The SNHG6 carcinogenicity was discussed in many investigations (28, 29). However, SNHG6 splice variants have not been addressed as much as deserved. Splice variants originate from the alternative splicing of a gene. The exons of a gene can be arranged along with each other in different arrangements and make different splice variants of that gene (30). The SNHG6 has eight splice variants that are the product of its alternative splicing. The SNHG6 203 was demonstrated as an oncogenic variant of this gene in hepatocellular carcinoma by Cao and colleagues (23). Many splice variants in lncRNAs have unknown functions. The splice variants of SNHG6 could have their expression pattern in distinct cancers. For example, SNHG6 203 and 207 in colorectal cancer show different expression patterns (24). 

We evaluated the expression level of four splice variants of SNHG6 in 47 patients with breast cancer and realized that SNHG6 203 was up-regulated significantly in tumor samples compared to non-tumors among all investigated variants. The SNHG6 203 was expressed in HER2-negative breast tumors more frequently than the HER2-positive cases (Figure 1). These data encouraged us to investigate the effects of SNHG6 203 overexpression in cell lines with different HER2 statuses. We chose MCF7 and SK-BR3 cell lines for our purpose. After the overexpression of SNHG6 203, we evaluated the impact of this transcript on the cell cycle progression, cell migration, and cell viability of target cells. 

The SNHG6 203 slightly stimulated the G1 progression of MCF7 cells. SK-BR3 overexpressing cells showed no G1 progression versus MCF7 ones (Figure 4). The cells we studied were different in the number of HER2 receptors on their surface. Abnormal HER2 signaling initiates a pathway that leads to irregular breast cancer cell proliferation (31, 32). Cell cycle progression of SNHG6 203 overexpressing MCF7 cells (as HER2-negative breast cancer cells) (Figure 4A) demonstrated that SNHG6 203, similar to lncRNA Miat, could be a helpful factor in cell cycle progression (26). The cell cycle of SNHG6 203 overexpressing SK-BR3 cells did not progress (Figure 4B) even though they were HER2-positive as a forte and this phenomenon could confirm our claim. 

Migration of SNHG6 203 overexpressing cells was different from their respective controls. After overexpression, the migration ability of the cells declined in both cell lines, and this reduction was sharper in MCF7 cells (Figure 5). MCF7 and SK-BR3 cells have their special cell-cell adhesions, invasion and migration characteristics (33, 34). Furthermore, target cells have their defined SNHG6 203 expression levels as a probable effective factor for regulating tight junction proteins (35). All mentioned items could explain different migration behaviors of MCF7 and SK-BR3 cells after SNHG6 203 overexpression. 

The MTT assay showed that the effect of SNHG6 203 on the viability of HER2-positive and -negative cells was different. MCF7 cells, as HER2-negative cells, indicated slight and non-significant viability difference after SNHG6 203 overexpression, while the viability of SK-BR3 overexpressing cells decreased more sharply than their respective controls (Figure 6). This reduction may result from SNHG6 203 interference in the metabolic pathways of these cells (36). Hormone receptors could be interconnected with lncRNAs expression in the cells and this relation could affect cellular metabolism (37). Moreover, in SNHG6 203 overexpression situation, the complicated behavior of MCF7 and SK-BR3 cells could be attributed to different hormonal signaling (38, 39). 

## Conclusion

Overall, the results of this study demonstrated an interconnection between SNHG6 203 RNA and HER2 status of breast tumoral cells that might indicate SNHG6 203 probable role in the cell cycle progression of HER2-negative breast cancers.

## Conflict of Interest

The authors declare no conflict of interests.

## Funding

The author(s) received no financial support for the research, authorship, and/or publication of this article.

## References

[1] Wang KC, Chang HY (2011). Molecular mechanisms of long noncoding RNAs. Mol Cell.

[2] Yao RW, Wang Y, Chen LL (2019). Cellular functions of long noncoding RNAs. Nat Cell Biol.

[3] Zampetaki A, Albrecht A, Steinhofel K (2018). Long Non-coding RNA Structure and Function: Is There a Link?. Front Physiol.

[4] Mathews DH, Turner DH, Zuker M (2007). RNA secondary structure prediction. Curr Protoc Nucleic Acid Chem.

[5] Huarte M (2015). The emerging role of lncRNAs in cancer. Nat Med.

[6] Mozdarani H, Ezzatizadeh V, Rahbar Parvaneh R (2020). The emerging role of the long noncoding RNA HOTAIR in breast cancer development and treatment. J Transl Med.

[7] Xie H, Liao X, Chen Z, Fang Y, He A, Zhong Y (2017). LncRNA MALAT1 Inhibits Apoptosis and Promotes Invasion by Antagonizing miR-125b in Bladder Cancer Cells. J Cancer.

[8] Lv P, Qiu X, Gu Y, Yang X, Xu X, Yang Y (2019). Long noncoding RNA SNHG6 enhances cell proliferation, migration and invasion by regulating miR-26a-5p/MAPK6 in breast cancer. Biomed Pharmacother.

[9] Cai G, Zhu Q, Yuan L, Lan Q (2018). LncRNA SNHG6 acts as a prognostic factor to regulate cell proliferation in glioma through targeting p21. Biomed Pharmacother.

[10] Yan K, Tian J, Shi W, Xia H, Zhu Y (2017). LncRNA SNHG6 is Associated with Poor Prognosis of Gastric Cancer and Promotes Cell Proliferation and EMT through Epigenetically Silencing p27 and Sponging miR-101-3p. Cell Physiol Biochem.

[11] Zimta AA, Tigu AB, Braicu C, Stefan C, Ionescu C, Berindan-Neagoe I (2020). An Emerging Class of Long Non-coding RNA With Oncogenic Role Arises From the snoRNA Host Genes. Front Oncol.

[12] Holley C, Elliot B, Ho HT (2019). Modification of mRNA by snoRNA‐guided 2′‐methylation. FASEB J..

[13] Stepanov GA, Filippova JA, Komissarov AB, Kuligina EV, Richter VA, Semenov DV (2015). Regulatory role of small nucleolar RNAs in human diseases. BioMed Res Int.

[14] Popis MC, Blanco S, Frye M (2016). Posttranscriptional methylation of transfer and ribosomal RNA in stress response pathways, cell differentiation, and cancer. Curr Opin Oncol.

[15] Jafari-Oliayi A, Asadi MH (2019). SNHG6 is upregulated in primary breast cancers and promotes cell cycle progression in breast cancer-derived cell lines. Cell Oncol.

[16] Meng Q, Yang BY, Liu B, Yang JX, Sun Y (2018). Long noncoding RNA SNHG6 promotes glioma tumorigenesis by sponging miR-101-3p. Int J Biol Markers.

[17] Yan Y, Chen Z, Xiao Y, Wang X, Qian K (2019). Long noncoding RNA SNHG6 is upregulated in prostate cancer and predicts poor prognosis. Mol Biol Rep.

[18] Geng H, Li S, Xu M (2020). Long Noncoding RNA SNHG6 Functions as an Oncogene in Non-Small Cell Lung Cancer via Modulating ETS1 Signaling. Onco Targets Ther.

[19] Wu Y, Deng Y, Guo Q, Zhu J, Cao L, Guo X (2019). Long noncoding RNA SNHG6 promotes cell proliferation and migration through sponging miR-4465 in ovarian clear cell carcinoma. J Cell Mol Med.

[20] Wang HS, Zhang W, Zhu HL, Li QP, Miao L (2020). Long noncoding RNA SNHG6 mainly functions as a competing endogenous RNA in human tumors. Cancer Cell Int.

[21] Xu M, Chen X, Lin K, Zeng K, Liu X, Xu X (2019). lncRNA SNHG6 regulates EZH2 expression by sponging miR-26a/b and miR-214 in colorectal cancer. J Hematol Oncol.

[22] Wang H, Wang L, Tang L, Luo J, Ji H, Zhang W (2020). Long noncoding RNA SNHG6 promotes proliferation and angiogenesis of cholangio-carcinoma cells through sponging miR-101-3p and activation of E2F8. J Cancer.

[23] Cao C, Zhang T, Zhang D, Xie L, Zou X, Lei L (2017). The long noncoding RNA, SNHG6-003, functions as a competing endogenous RNA to promote the progression of hepatocellular carcinoma. Oncogene.

[24] Jafari Oliayi A, Asadi MH (2018). SNHG6 203 and SNHG6 201 Transcripts Can be Used as Contributory Factors for a Well-Timed Prognosis and Diagnosis of Colorectal Cancer. J Kerman Uni Med Sci.

[25] Schmittgen TD, Livak KJ (2008). Analyzing real-time PCR data by the comparative CT method. Nat Protoc.

[26] Alipoor FJ, Asadi MH, Torkzadeh-Mahani M (2018). MIAT lncRNA is overexpressed in breast cancer and its inhibition triggers senescence and G1 arrest in MCF7 cell line. J Cell Biochem.

[27] Keshavarz M, Asadi MH (2019). Long noncoding RNA ES1 controls the proliferation of breast cancer cells by regulating the Oct4/Sox2/miR-302 axis. FEBS J.

[28] Lan Z, Yao X, Sun K, Li A, Liu S, Wang X (2020). The Interaction Between lncRNA SNHG6 and hnRNPA1 Contributes to the Growth of Colorectal Cancer by Enhancing Aerobic Glycolysis Through the Regulation of Alternative Splicing of PKM. Front Oncol.

[29] Teppan J, Barth DA, Prinz F, Jonas K, Pichler M, Klec C (2020). Involvement of Long Non-Coding RNAs (lncRNAs) in Tumor Angiogenesis. Noncoding RNA.

[30] El Marabti E, Younis I (2018). The Cancer Spliceome: Reprograming of Alternative Splicing in Cancer. Front Mol Biosci.

[31] Lissoni P, Messina G, Rovelli F, Brivio F, Fumagalli L, Villa S (2009). HER2 expression in breast cancer: correlation with endocrine function and psychological status in operable and metastatic breast cancer. In Vivo.

[32] Huang F, Shi Q, Li Y, Xu L, Xu C, Chen F (2018). HER2/EGFR-AKT Signaling Switches TGFbeta from Inhibiting Cell Proliferation to Promoting Cell Migration in Breast Cancer. Cancer Res.

[33] Liu YL, Chou CK, Kim M, Vasisht R, Kuo YA, Ang P (2019). Assessing metastatic potential of breast cancer cells based on EGFR dynamics. Sci Rep.

[34] Oberst M, Anders J, Xie B, Singh B, Ossandon M, Johnson M (2001). Matriptase and HAI-1 are expressed by normal and malignant epithelial cells in vitro and in vivo. Am J Pathol.

[35] Xiao L, Rao JN, Cao S, Liu L, Chung HK, Zhang Y (2016). Long noncoding RNA SPRY4-IT1 regulates intestinal epithelial barrier function by modulating the expression levels of tight junction proteins. Mol Biol Cell.

[36] Zhao XY, Lin JD (2015). Long Noncoding RNAs: A New Regulatory Code in Metabolic Control. Trends Biochem Sci.

[37] Lin A, Li C, Xing Z, Hu Q, Liang K, Han L (2016). The LINK-A lncRNA activates normoxic HIF1alpha signalling in triple-negative breast cancer. Nat Cell Biol.

[38] Ottaviani S, de Giorgio A, Harding V, Stebbing J, Castellano L (2014). Noncoding RNAs and the control of hormonal signaling via nuclear receptor regulation. J Mol Endocrinol.

[39] Boone DN, Warburton A, Som S, Lee AV (2020). SNHG7 is a lncRNA oncogene controlled by Insulin-like Growth Factor signaling through a negative feedback loop to tightly regulate proliferation. Sci Rep.

